# Do modern family planning methods impact women’s quality of life? Jordanian women’s perspective

**DOI:** 10.1186/s12955-019-1226-6

**Published:** 2019-10-15

**Authors:** Mohammad S. Alyahya, Heba H. Hijazi, Hussam A. Alshraideh, Nihaya A. Al-sheyab, Dana Alomari, Sara Malkawi, Sarah Qassas, Samah Darabseh, Yousef S. Khader

**Affiliations:** 1 Department of Health Management and Policy, Faculty of Medicine, Jordan University of Science and Technology, P.O.Box 3030, Irbid, 22110 Jordan; 2 Industrial Engineering, Faculty of Engineering, Jordan University of Science and Technology, P.O.Box (3030), Irbid, 22110 Jordan; 3 Allied Medical Sciences Department/Faculty of Applied Medical Sciences, Jordan University of Science and Technology, P.O.Box (3030), Irbid, 22110 Jordan; 4 Medical Education and Biostatistics, Department of Community Medicine, Public Health and Family Medicine, Jordan University of Science & Technology, Irbid, 22110 Jordan

**Keywords:** Quality of life, QoL, Contraceptives, Family planning methods, WHOQOL-BREF, Jordan

## Abstract

**Background:**

Although Jordan has made progress in meeting Family Planning (FP) needs in last decades, recently the use of FP methods has declined significantly. Women’s personal experiences, knowledge, and perceptions of how a FP method might impact their quality of life (QoL) can influence FP decisions. However, a lack of comprehensive understanding of the impact of modern FP methods on women’s QoL continues to exist among Jordanian couples. Therefore, this study aimed to investigate the relationship between the use of common modern FP methods and QoL among Jordanian women.

**Methods:**

Using the WHOQOL-BREF questionnaire along with other questions, non-pregnant women of reproductive age were interviewed at their homes through face-to-face structured interviews. Women who visited the obstetrics and gynecology clinic of King Abdullah University Hospital for contraceptive advice and follow-up consultations were also included.

**Results:**

A total of 548 women aged between 18 and 49 participated in the study. Based on the WHOQOL-BREF scale, the overall mean (SD) scores of the four domains were found to be average. Our findings show that women who used Intra Uterine Devices (IUDs) and women whose husbands used condoms had better QoL in the four domains (physical health, psychological health, social relationships, and environment) than those who used Oral Contraceptives (OCs). Women who used implant and injectable hormonal contraceptives had better QoL in terms of the physical health and social relationships domains. In contrast, women who had undergone permanent sterilization had lower QoL scores in all of the four domains. Further analysis revealed that women who had undergone tubal sterilization were less satisfied overall and more likely to experience side effects than women who used OCs.

**Conclusion:**

The choice to use contraceptives and decide freely whether and when to have children is regarded as a fundamental reproductive health right and is strongly linked to women’s health and QoL. Women who use OCs and women who have undergone permanent sterilization are likely to have lower QoL than women who use IUDs or implant and injectable hormones and those whose husbands use condoms.

## Background

Modern family planning (FP) methods have become more broadly accepted and used as a replacement for traditional methods and as one of the interventions to most empower women. Women empowerment, as an important goal for achieving national development goals, needs to be strengthened and sustained to ensure that women exercise their responsibilities and enjoy their human rights [1–3]. Women status and empowerment have been found to be associated with longer birth interval, lower rate of unintended pregnancy, and lower fertility rate (FR) [3]. Through FP programs, women have been given access to modern contraceptives, allowing them to better meet their fertility preferences and to avoid unwanted pregnancies and their potential complications [4–7]. Nonetheless, even in highly developed countries such as the U.S., unintended pregnancies remain high [8] and constitute a health and socio-economic burden for women and their partners [9, 10]. An obvious explanation is that there remain socio-cultural barriers and perceived health risks associated with the use of new FP methods [11–13].

Previous research has shown that the main challenges facing women and preventing them from using modern contraceptive methods include the social pressure on women to prove their fertility immediately after marriage and the fear of side effects and negative consequences of modern FP methods [14–16]. In a recent qualitative study involving 42 focus group discussions with Jordanian and Syrian young couples, there was a strong belief among participants that most modern FP methods can have serious side effects that could harm women’s health, including bleeding, hypertension, diabetes, foetal abnormalities, and cancer [17]. These concerns often drive married couples to use less effective but safer methods, such as withdrawal and condoms.

Women’s personal experiences, knowledge, and perceptions of how a FP method might impact their quality of life (QoL) can influence FP decisions among women. While different FP methods may have varying degrees of impact on women’s health-related quality of life (HRQoL) [18], there yet remains no consensus on the relationship between modern FP methods and the QoL of women. Most of the previous research has focused on either single or limited types of contraceptives and on only one or two QoL domains (i.e. sexual and psychological) [19–25]. For instance, it has been found that combined oral pills statistically decreased general well-being among healthy women and reduced three of the six dimensions of the Psychological General Well-Being Index (PGWBI), namely positive well-being, self-control, and vitality [19]. The authors concluded that even a modest reduction in general well-being could be of importance and could explain the high incompliance rate and irregular use of oral contraceptives (OCs) [19]. Similarly, Sadatmahalleh et al. observed lower sexual function and QoL in women who had undergone tubal ligation than in women who had not, hence concluding that tubal ligation is not a safe FP method [26]. In contrast, intrauterine contraception has been shown to significantly improve HRQoL [20]. On the other hand, a recent systematic review by Worly et al. revealed that the available studies do not support a clear general association between progestin hormonal contraceptives and depression levels or incidence of depression diagnosis [25].

### Modern FP use among Jordanian women

Although the Total Fertility Rate (TFR) in Jordan has declined steadily in recent decades to reach 3.5 in 2014, it has not changed significantly during the last few years [27]. Jordan has made some progress in meeting FP needs, such that the use of modern contraceptives increased by 15% from 1990 to 2012 [28]. However, the use of both modern and traditional FP methods has declined from 42 to 37%, and from 19 to 14% between 2012 and 2017/2018, respectively. Among currently married women adopt a modern method of FP, the most commonly used method is the Intra Uterine Devices (IUDs) (56%), followed by the pill (21%), and the male condom (13%) [28, 29]. The majority of modern contraceptive methods are accessible to women through the primary and comprehensive healthcare centers of the Ministry of Health. Oral contraceptives, Etonogestrel implants, Depo Provera contraceptive injections, male condoms, and IUDs are offered free of charge, and there are some facilities for female sterilization.

The process of choosing FP methods in Jordan is primarily influenced by the husband’s decision and preference. About 85% of women currently using contraceptives report that the decision to use any FP method was a shared decision with the husband. Additionally, two thirds (77%) of those who report not using any method say that the decision not to use was also a mutual decision with the husband, regardless of the education level. Women reported that they first discussed FP and the likelihood of using contraception with husbands, doctors and midwives, and friends. Oral contraceptives and IUDs were the two most common methods suggested by those advisers. When women decide to start using contraception, physicians were the most sought person to give advice as they are highly trusted by women [29, 30]. However, a lack of comprehensive understanding of the impact of modern FP methods continues to exist among Jordanian couples.

To date, research addressing the impact of different modern FP methods on women’s QoL remains limited [31–34]. A study found that women who had undergone tubal ligation and those who used injectable contraceptive were less likely to have average or better physical HRQoL than women who used combined hormonal contraceptives and that women who used injectable contraceptive were also less likely to have average or better mental-HRQoL [18]. Another study showed that women who had undergone tubal ligation had significantly worse “physical functioning” but better “general health perceptions” than women whose partners used condoms [32].

To this end, the primary aim of this study was to examine the relationship between the use of common modern FP methods and QoL among Jordanian women. Also, identifying main predictors of QoL was another aim of the study.

## Methods

### Study design and setting

A cross-sectional household survey in two cities in Jordan was conducted from April to December 2017. Each city was stratified into 5 sectors based on geographical location and density of population. Housewives from each sector were visited in their houses, and working women who were unavailable during the morning were visited in the afternoon. An initial screening (short-structured interviews) was first conducted to ensure that each woman was eligible and that she consented to participation in the study. The screening questions included information on age, health status, medical history, and whether the participant was pregnant. If the woman was eligible, the female interviewers explained to her the objectives of the study and assured her of the confidentiality of the interview before commencing with the interview itself. Talking about family planning methods is a socio-cultural sensitive issue in Jordan, especially among young women. Many Jordanian women usually feel embarrassed to discuss family planning methods, and this led to a low response rate of around 15% of the household survey. For this reason, non-pregnant women who visited the obstetrics and gynecology clinic of King Abdullah University Hospital (KAUH) for contraceptive advice and follow-up consultations were also included. KAUH is a teaching referral hospital that provides a wide range of services. Ethical approval was sought and obtained from the Institutional Research Committee of Jordan University of Science and Technology. After full clarification of the study aims and ethical considerations, any woman who showed interest in participating and gave consent was included. Since contraception is a culturally sensitive issue in Jordan, willingness to participate voluntarily was a main inclusion criterion, and thus, a convenience sampling method was applied.

### Data collection and measures

According to the latest national survey, about 30% of Jordanian women discontinued using the method within 12 months. The most common reason for discontinuations was the desire to become pregnant (54%), followed by complaints that the method was inconvenient to use (12%) [29]. Consequently, non-pregnant women of child bearing age (18–49) who had been using a modern contraceptive method (the same method) for the past year were interviewed through face-to-face structured interviews. Three female researchers were trained to conduct the interviews, where each one was responsible for conducting the interviews in a particular geographical location/area. A structured interview questionnaire guide (in Arabic) composed of four main sections was used for data collection. The socio-demographic data form included questions about the woman’s age, number of living children, age at marriage, and religion. The first section also included statements on socioeconomic status, including educational attainment, employment class, monthly income, housing tenure, and the number of dependents. The second section included questions on birth spacing and on the type, duration of use, and previous information of the modern FP methods used, as well as previous knowledge of their side effects. Another questionnaire, namely the Arabic version of World Health Organization Quality of Life-BREF (WHOQOL-BREF), was used as the measuring instrument for QoL in the current study.

The WHOQOL-BREF is a cross cultural, well validated, generic questionnaire that has been employed in various clinical conditions in different countries around the globe [31, 35–37]. According to the WHO report [38], the WHOQOL-BREF serves as an adequate alternative instrument to the evaluation of domain profiles using WHOQOL-100. With a total of 26 questions, the instrument contains four domains, namely physical health, psychological, social relationships, and environment. These questions use a five point Likert response scale. There are also two questions in the WHOQOL-BREF that are evaluated separately; the first question asks about the participant’s overall perception of their QoL and the second about the participant’s overall perception of their health [39].

While the WHOQOL-BREF examines respondents’ perceived QoL and can thus provide an overview of the participant’s perceived effects of disease and health interventions, it is not supposed to act as an objective tool for measuring symptoms and disability. Therefore, the WHOQOL is “an assessment of a multi-dimensional concept incorporating the individual’s perception of health status, psycho-social status, and other aspects of life.” [38].

As per the WHO guidelines, the mean score of questions within each domain is used to calculate the score of the domain; means are multiplied by 4 and subsequently transformed into a 0 to 100 scale [38]. The domain scores are comparable with the scores used in the WHOQOL-100, and the mean score in each domain reflects women’s perceptions of and satisfaction with each aspect of their quality of life. The higher the score, the better the response. A permission to use the WHOQOL-BREF was obtained from the Information, Evidence, and Research (IER) Department / The World Health Organization, Geneva. The last section of the interview guide contained questions about side effects of modern FP methods and bleeding patterns. At the end of each interview, the participating woman was asked to read and check the filled data for accuracy, and then she was voluntarily asked to sign the filled questionnaire. Each interview lasted about 20 min.

### Data analysis

Data was analyzed using R Statistical Computing Software version 3.4.3 (R Foundation for Statistical Computing, Vienna, Austria). Descriptive statistics including means, standard deviations, and percentages for collected data are presented. QoL scores for the four domains were calculated individually for each participating woman, and then the total scores for each domain were summarized. Finally, predictors of quality of life scores were identified through multiple linear regression and stepwise selection. A 5% significance level was assumed throughout the analysis.

## Results

Five hundred forty-eight women aged between 18 and 49 (mean age 34.7, SD 6.14) participated in the study. The participating women had been using a FP method for an average of 5.17 (SD 2) years, discontinuously. The results show that the average time from marriage to first pregnancy was 3.31 (SD 2.36) months, and the average number of children per family was 3.79 (SD 1.85). The mean duration of spacing between births was 2.78 (SD 1.17) years. Detailed information on the participants is provided in Table 1. Around one third of the participants (35.6%) had a graduate or post-graduate academic degree, around 33% had a family income less than 400 JDs, and 58.5% resided in rented accommodation. In Jordan, there is no clear official definition of the poverty line. However, when taking into consideration the latest available data and reports, an estimated family income of (JD400) per month can be considered as the poverty line [40, 41]. In accordance with the national figures, about 9.5% of the participants were smokers [42], and only 11.86% were employed [43]. The results also show that about 9.52% of the participating women used a FP method without a doctor’s prescription. Unfortunately, about 80.66% of the women had no previous knowledge of the possible side effects of using FP methods, and only 46.72% of them had had enough information about FP methods before starting to use them.

**Table 1:** Summary of participants’ information

Factor	Categories	Oral contraceptives	IUD	Implant and injectable hormonal contraceptives	Sterilization	Male condoms	Total
Education level	Illiterate	0.78%	2.19%	2.46%	0	7.69%	2.55%
	Primary school	22.04%	18.42%	12.34%	17.64%	19.23%	18.43%
	Secondary school	37.79%	35.52%	35.8%	17.64%	34.61%	34.85%
	High school	7.08%	8.33%	7.40%	20.58%	8.97%	8.75%
	Graduate/Post Graduate	32.28%	35.52%	41.97%	29.14%	29.48%	35.58%
Income	Less than 400 JD	35.43%	27.19%	35.80%	41.17%	30.76%	32.52%
	More than 400 JD	62.20%	70.61%	60.49%	58.82%	65.38%	59.85%
Residency	Rent	58.26%	54.38%	69.13%	67.64%	56.41%	58.57%
	Owned	40.94%	44.29%	28.39%	20.58%	38.46%	38.86%
	Other	0.78%	1.31%	2.46%	11.76%	3.84%	2.37%
Smoking	No	88.97%	91.6%	96.29%	85.29%	85.89%	91.61%
	Yes	11.02%	8.33%	3.70%	14.70%	14.10%	9.49%
Employment status	Employed	10.23%	7.45%	18.51%	20.58%	26.92%	11.86%
	Unemployed	89.76%	92.54%	81.48%	79.41%	73.07%	88.13%
Prescribed FP	No	10.24%	10.13%	9.88%	8.82%	6.49%	9.52%
	Yes	89.76%	89.87%	90.12%	91.18%	93.51%	90.48%
Prior knowledge of side effects	Yes	11.81%	22.81%	25.93%	20.59%	14.1%	19.34%
	No	88.19%	77.1%9	74.07%	79.41%	85.9%	80.66%
Had enough information about FP	Yes	46.46%	44.3%	54.32%	41.18%	48.72%	46.72%
	No	53.54%	55.7%	45.68%	58.82%	51.28%	53.28%
Noticed bleeding	Always	11.11%	1.32%	13.75%	0	8.97%	6.42%
	Very often	35.71%	17.62%	42.5%	20.59%	33.33%	27.89%
	Quite often	33.33%	25.11%	36.25%	17.65%	26.92%	28.44%
	Seldom	11.9%	27.31%	5%	20.59%	17.95%	18.72%
	Never	7.94%	28.63%	2.5%	41.18	12.82%	18.53%
Self-reported QoL	Very poor	1.57%	1.75%	4.94%	47.06%	5.13%	5.47%
	Poor	3.15%	4.82%	4.94%	32.35%	11.54%	7.12%
	Neither poor nor good	48.82%	34.65%	43.21%	5.88%	34.62%	37.41%
	Good	35.43%	44.3%	30.86%	5.88%	38.46%	37.04%
	Very good	11.02%	14.47%	16.05%	8.82%	10.26%	12.96%
Self-reported health satisfaction	Very dissatisfied	1.57%	3.07%	1.23%	38.24%	5.13%	4.93%
	Dissatisfied	14.96%	10.53%	18.52%	26.47%	14.1%	14.23%
	Neither satisfied nor dissatisfied	40.94%	46.05%	33.33%	26.47%	33.33%	39.96%
	Satisfied	42.52%	36.4%	46.91%	5.88%	35.9%	37.41%
	Very satisfied	0	3.95%	0	2.94%	11.54%	3.47%

As for the types of contraceptive methods used, 41.61% of the participants used IUDs, 23.18% used oral contraceptives, 14.78% used implant and injectable hormonal contraceptives, 6.2% used sterilization, and 14.23% reported that their husbands used condoms as a FP method. Only about 50% rated their QoL as being good or very good and about 40.88% were satisfied with their health status.

Women’s responses to QoL related questions of the WHOQOL-BREF tool are summarized in Table 2. Based on these responses, scores for the four QoL domains, namely physical health, psychological, social relationships, and environment, were calculated for each woman. A summary of these domain scores, along with the FP method used, is shown in Table 3. From highest to lowest, average scores for the social relationships, psychological, physical health, and environmental domains were 13.32, 12.97, 12.94, and 12.81, respectively, on a scale of 4–20. Women who used IUDs had the highest QoL average score for the physical health domain, while those whose husbands used condoms had a much higher average QoL scores for the psychological, social relationships, and environment domains than other women’s groups.

**Table 2:** Percentages of participants’ responses for the quality of life related questions

Question		Not at all (%)	A little (%)	A moderate amount (%)	Very much (%)	An extreme amount (%)
Q3	To what extent do you feel that physical pain prevents you from doing what you need to do?	11.9	31.8	42.0	10.0	4.4
Q4	How much do you need any medical treatment to function in your daily life?	12.4	30.3	37.6	12.6	7.1
Q5	How much do you enjoy life?	3.8	10.9	46.7	28.5	10.0
Q6	To what extent do you feel your life to be meaningful?	2.4	12.4	42.2	27.4	15.7
Q7	How well are you able to concentrate?	4.6	14.8	45.6	24.8	10.2
Q8	How safe do you feel in your daily life?	4.9	13.3	35.2	32.3	14.2
Q9	How healthy is your physical environment?	4.4	17.5	46.2	20.6	11.3
Q10	Do you have enough energy for everyday life?	8.2	27.9	42.0	16.1	5.8
Q11	Are you able to accept your bodily appearance?	4.6	14.1	39.8	26.8	14.8
Q12	Have you enough money to meet your needs?	2.0	17.2	48.4	24.8	7.7
Q13	How available to you is the information that you need in your day-to-day life?	5.3	20.7	38.0	28.4	7.5
Q14	To what extent do you have the opportunity for leisure activities?	6.6	18.8	41.1	23.0	10.6
		Very bad	Bad	Okay	Good	Very good
Q15	How well are you able to get around?	5.3	12.4	18.2	14.8	49.3
		Very dissatisfied	Dissatisfied	Neither satisfied nor dissatisfied	Satisfied	Very satisfied
Q16	How satisfied are you with your sleep?	7.5	19.5	23.9	35.0	14.1
Q17	How satisfied are you with your ability to perform your daily living activities?	11.9	16.6	19.7	30.8	21.0
Q18	How satisfied are you with your capacity for work?	23.5	21.0	26.8	24.3	4.4
Q19	How satisfied are you with yourself?	7.5	13.5	29.4	35.6	14.1
Q20	How satisfied are you with your personal relationships?	4.2	10.4	34.5	35.2	15.7
Q21	How satisfied are you with your sex life?	5.8	15.9	37.0	25.5	15.7
Q22	How satisfied are you with the support you get from your friends?	3.3	16.8	37.6	32.1	10.2
Q23	How satisfied are you with the conditions of your living place?	2.9	14.1	41.4	32.5	9.1
Q24	How satisfied are you with your access to health services?	10.8	25.0	34.7	20.6	8.9
Q25	How satisfied are you with your transport?	5.8	15.7	30.1	27.6	20.8
		Always	Very often	Quite often	Seldom	Never
Q26	How often do you have negative feelings such as blue mood, despair, anxiety, depression?	14.1	17.3	27.7	20.3	20.6

**Table 3:** Summary of domain scores. Statistics are based on 4–20 scale

FP method	Physical health		Psychological		Social relationships		Environment	
	Mean	SD	Mean	SD	Mean	SD	Mean	SD
OCPs	12.21	2.56	12.70	1.83	13.09	2.81	12.32	2.00
IUD	13.77	1.90	12.89	2.14	13.32	3.03	13.11	2.05
Implant and injectable hormonal contraceptives	12.83	1.94	12.69	2.12	13.32	2.44	12.24	2.06
Sterilization	11.58	1.96	10.35	1.82	10.63	3.22	9.71	1.90
Male condoms	12.42	2.35	15.30	2.26	15.57	2.78	14.70	2.46
Overall	12.94	2.26	12.97	2.29	13.42	3.06	12.81	2.37

Multiple linear regression in combination with the stepwise selection method was used to identify the significant predictors of the domain scores and to identify the effect of FP methods on the QoL of the participating women. As the effect of the FP method used might be confounded with other variables such as the socioeconomic status and age, all collected variables were used in the regression model to adjust for the effects of the FP methods. Significant predictors along with the model coefficients for the four domains are shown in Table 4. Multicollinearity among used predictors was assessed through variance inflation factors (VIF). Values for VIF were all less than 10 indicating no multicollinearity issues. Significant predictors of physical health scores were level of education, income, residency, employment status, birth spacing, side effects, type of contraception, quality of life rating, and satisfaction with health. In comparison to women who used OCs, women who used IUDs for family planning had the highest probability of good physical health scores (β = 1.29), followed by women who use implants and injectable hormonal contraceptives (β = 0.66), whereas those who used sterilizations were more likely to score low in the physical health score (β = − 0.99).

**Table 4:** Predictors of the four QoL domains

*1-* Predictors of the physical health domain scores.		*β*	*95% CI*	*p*
	(Intercept)	11.78	9.58–13.98	<.001
Education	*Primary school*	2.81	0.79–4.84	0.007
	*Secondary school*	0.37	−1.43 – 2.16	0.688
	*Diploma*	1.48	−0.07 – 3.03	0.061
	*Bachelor’s degree*	0.66	−1.00 – 2.33	0.435
Income	*More than 400 JD*	0.73	0.35–1.11	<.001
Residency	*Owned*	0.18	−0.20 – 0.57	0.347
	*Other*	−1.1	−2.24 – 0.04	0.059
Employment status	*Employed*	0.79	0.17–1.42	0.012
Birth spacing		−0.16	− 0.31 – − 0.01	0.04
Experienced side effect after using FP	*Very often*	0.42	−0.14 – 0.97	0.141
	*Quite often*	0.46	−0.08 – 0.99	0.092
	*Seldom*	1.08	0.45–1.72	<.001
	*Never*	0.76	0.11–1.42	0.023
Type of contraception used	*IUD*	1.29	0.79–1.79	<.001
	*Implant and injectable hormonal contraceptives*	0.66	0.08–1.24	0.026
	*Sterilization*	−0.99	−1.97 – 0.00	0.05
	*Male condoms*	0.1	−0.51 – 0.71	0.746
Self-reported QoL	*Poor*	−1.39	−2.44 – − 0.35	0.009
	*Neither poor nor good*	−0.82	−1.74 – 0.10	0.079
	*Good*	−0.11	−1.03 – 0.81	0.816
	*Very good*	0	−1.00 – 1.00	0.997
Self-reported health satisfaction	*Dissatisfied*	−1.15	−2.12 – −0.17	0.021
	*Neither satisfied nor dissatisfied*	−1.17	−2.10 – −0.23	0.015
	*Satisfied*	−0.75	−1.70 – 0.20	0.123
	*Very satisfied*	−1.73	−3.10 – − 0.36	0.013
R^2^		0.264		
*2-* Predictors of the psychological domain scores		*β*	*95% CI*	*p*
(Intercept)		12.49	10.55–14.42	<.001
Age		−0.04	−0.08 – 0.00	0.054
Education	*Primary school*	2.52	0.55–4.49	0.012
	*Secondary school*	2.31	0.58–4.04	0.009
	*Diploma*	1.8	0.31–3.29	0.018
	*Bachelor’s degree*	1.53	−0.14 – 3.21	0.072
Number of children		0.34	0.07–0.60	0.012
Time of first pregnancy		−0.05	−0.12 – 0.02	0.159
Birth spacing		−0.22	−0.37 – − 0.07	0.003
Experienced side effect after using FP	*Very often*	0.29	−0.25 – 0.83	0.287
	*Quite often*	0.28	−0.24 – 0.79	0.289
	*Seldom*	0.05	−0.56 – 0.65	0.879
	*Never*	−0.61	−1.25 – 0.02	0.059
Type of contraception used	*IUD*	0.05	−0.44 – 0.54	0.849
	*Implant and injectable hormonal contraceptives*	−0.14	−0.71 – 0.43	0.635
	*Sterilization*	−2.34	−3.25 – −1.43	<.001
	*Male condoms*	2.62	2.03–3.21	<.001
Self-reported QoL	*Poor*	−0.08	−1.06 – 0.90	0.87
	*Neither poor nor good*	−0.57	−1.45 – 0.30	0.198
	*Good*	−0.14	−1.03 – 0.74	0.751
	*Very good*	0.2	−0.75 – 1.15	0.684
R^2^		0.312		
*3-* Predictors of the social relationships domain scores		*β*	*95% CI*	*p*
(Intercept)		16.21	14.10–18.32	<.001
Age		−0.05	−0.10 – − 0.00	0.039
Income	*More than 400 JD*	0.53	−0.03 – 1.09	0.062
Employment status	*Employed*	0.71	−0.01 – 1.43	0.053
Birth spacing		−0.24	−0.44 – − 0.03	0.024
Type of contraception used	*IUD*	0.24	−0.40 – 0.88	0.465
	*Implant and injectable hormonal contraceptives*	0.32	−0.49 – 1.13	0.439
	*Sterilization*	−2.63	−3.80 – −1.46	<.001
	*Male condoms*	2.11	1.28–2.94	<.001
Self-reported health satisfaction	*Dissatisfied*	−1.54	−2.87 – −0.22	0.022
	*Neither satisfied nor dissatisfied*	−1.49	−2.75 – −0.24	0.02
	*Satisfied*	−0.86	−2.12 – 0.41	0.185
	*Very satisfied*	1.12	−0.71 – 2.95	0.23
R^2^		0.179		
*4-* Predictors of the environmental domain scores.		*β*	*95% CI*	*p*
(Intercept)		14.67	12.85–16.49	<.001
Age		−0.04	−0.09 – − 0.00	0.049
Income	*More than 400 JD*	0.98	0.58–1.38	<.001
Smoking	*Yes*	0.48	−0.15 – 1.10	0.135
Employment status	*Employed*	0.94	0.41–1.48	<.001
Duration of using FP		0.1	−0.02 – 0.22	0.113
Birth spacing		−0.15	−0.30 – − 0.01	0.04
Type of contraception used	*IUD*	0.7	0.24–1.15	0.003
	*Implant and injectable hormonal contraceptives*	−0.14	−0.71 – 0.44	0.641
	*Sterilization*	−3.21	−4.16 – −2.27	<.001
	*Male condoms*	2.08	1.49–2.67	<.001
Self-reported QoL	*Poor*	−0.37	−1.40 – 0.65	0.476
	*Neither poor nor good*	−0.71	−1.62 – 0.20	0.127
	*Good*	−0.05	−0.96 – 0.86	0.917
	*Very good*	0.65	−0.33 – 1.62	0.193
Self-reported health satisfaction	*Dissatisfied*	−1.15	−2.11 – −0.19	0.019
	*Neither satisfied nor dissatisfied*	−1.4	−2.32 – −0.49	0.003
	*Satisfied*	−1.39	−2.33 – −0.46	0.004
	*Very satisfied*	−0.54	−1.87 – 0.79	0.424
R^2^		0.312		

Level of education, number of children, birth spacing, side effects, contraceptive method, and QoL rating were the significant predictors of the psychological domain scores. Similar to the physical health scores, a negative effect was observed for sterilization (β = − 2.34), while positive effects were observed for IUDs (β = 0.05) and male condoms (β = 2.62). For the social relationships domain, age, income, occupation, birth spacing, contraceptive method, and satisfaction with health status were identified as the significant predictors. In comparison to women who used OCs, women who used IUDs (β = 0.24) or implants and injectable hormonal contraceptives (β = 0.32) and those whose husbands used condoms (β = 2.11) were more likely to have better scores, while those who used sterilization were more likely to score low in the social relationship (β = − 2.63). Finally, age, income, smoking, occupation, period of contraceptive use, birth spacing, contraceptive method, quality of life rating, and satisfaction with health status were the significant predictors of the environmental domain scores. For the environmental domain, women who used IUDs (β = 0.7) and those whose husbands used condoms (β = 2.08) as FP methods reported higher scores than women who used OCs. However, women who had received permanent sterilization had the worst environmental scores among all of the family planning methods (β = − 3.21).

To further investigate the effect of FP methods on QoL, multinomial logistic regression was used to identify significant predictors of side effects frequency, bleeding frequency, QoL rating perceptions, and satisfaction with health status rating. Identified significant predictors are shown in Table 5. Results indicate that the type of contraceptive method used was a key predictor of side effects, bleeding, QoL perception, and health satisfaction. Table 5 shows that women who had undergone sterilization reported the highest probability of frequent side effects while those whose husbands used condoms had a high probability of experiencing rare or no side effects. Women who used implant and injectable hormonal contraceptives had a very low probability of never experiencing side effects. Similarly, women who had undergone sterilization and those who used IUDs had the highest probability of bleeding. Results also show that women who used IUDs had the highest probability of good/very good quality of life perceptions while sterilized women had the lowest probability of good/very good perceptions.

**Table 5:** Predictors of side effect, bleeding frequency, quality of life rating perceptions, and health satisfaction

Predictors of side effects frequency		*Very often coefficients (β)*	*Quite often coefficients (β)*	*Seldom coefficients (β)*	*Never coefficients (β)*
(Intercept)		−0.235	−0.206	−1.564	−2.375
Type of contraception used	*IUD*	0.037	1.752	2.969	3.953
	*Implant and injectable hormonal contraceptives*	0.117	−0.046	0.214	−12.639
	*Sterilization*	1.844	2.151	3.760	4.859
	*Male condoms*	0.493	0.317	0.928	1.621
R^2^		0.096			
Predictors of bleeding frequency		*Very often coefficients (β)*	*Quite often coefficients (β)*	*Seldom coefficients (β)*	*Never coefficients (β)*
(Intercept)		0.767	−0.400	−0.683	−1.422
Number of kids		0.200	0.660	0.360	0.497
Type of contraception used	*IUD*	1.321	1.673	2.754	3.221
	*Implant and injectable hormonal contraceptives*	−0.072	−0.250	−1.144	−1.461
	*Sterilization*	12.473	12.405	13.553	14.661
	*Male condoms*	0.069	−0.121	0.435	0.347
R^2^		0.079			
Predictors of quality of life rating perceptions		*Poor coefficients (β)*	*Neither poor nor good coefficients (β)*	*Good coefficients (β)*	*Very good coefficients (β)*
(Intercept)		4.291	6.855	6.681	0.992
Age		−0.088	−0.247	−0.252	−0.215
Occupation	*Employed*	1.077	2.652	2.227	−7.784
Number of children		−1.334	0.516	0.492	0.088
Type of contraception used	*IUD*	0.351	0.107	0.608	0.751
	*Implant and injectable hormonal contraceptives*	−0.364	−0.600	−0.730	−0.086
	*Sterilization*	−1.435	−5.528	−5.308	−3.771
	*Male condoms*	0.189	−1.003	−0.633	−0.706
R^2^		0.135			
Predictors of health satisfaction rating		*Dissatisfied coefficients (β)*	*Neither satisfied nor dissatisfied coefficients (β)*	*Satisfied coefficients (β)*	*Very satisfied coefficients (β)*
(Intercept)		16.524	37.553	35.287	14.479
Age		−0.119	−0.218	−0.112	− 0.268
Education	*Primary school*	−1.470	12.234	−22.899	31.520
	*Secondary school*	13.002	−15.804	−16.384	−26.168
	*Diploma*	12.966	−14.480	−14.109	− 14.464
	*Bachelor’s degree*	3.744	28.977	28.734	31.989
Income	More than 400 JD	8.418	19.101	17.793	8.403
Number of children		1.164	1.145	1.227	0.398
Prescribed FP	Yes	−35.251	−34.918	−34.166	−35.935
Years of using FP		0.133	0.113	−0.115	−0.300
Type of contraception used	*IUD*	−0.933	−0.098	− 0.689	36.992
	*Implant and injectable hormonal contraceptives*	−2.147	−2.904	−4.713	35.440
	*Sterilization*	0.637	0.398	0.439	4.760
	*Male condoms*	−0.951	−0.997	−1.254	37.529
R^2^		0.158			

Women who used IUDs or implant and injectable hormonal contraceptives and women whose husbands used condoms had the highest level of health satisfaction, while women who used oral pills and sterilization had the lowest health satisfaction.

## Discussion

From social, psychological, and health-related viewpoints, FP methods are of great importance in women’s lives [44, 45]. This study investigated the relationship between the use of common modern FP methods and QoL domains among Jordanian women. In line with the latest Jordanian national survey 2017/2018 [29], our findings observed that the most commonly used modern FP method is the IUDs, followed by OCs. The findings also revealed that about a tenth of Jordanian women of reproductive age had previously used a modern FP method without medical prescriptions or advice. Furthermore, more than half of the participating women did not have enough previous information about modem FP methods. It has been found recently that the percentage of Jordanian women who were not informed about side effects of modern FP methods ranged between 20 to 39% among various methods including pills, female sterilization, IUD, implants and injectables [29]. Nevertheless, women who intend to use modern FP methods have the right to know the potential side effects and negative consequences of these methods, including emotional, psychological, physiological, and sexual consequences.

Most women in developing countries, including Jordan, prefer to get pregnant immediately after marriage [46–49], and hence an average time from marriage till first pregnancy of only 3.31 months was observed among the participating women. Often, this short waiting period is a result of social pressure and reflects the tendency among young women to prove their fertility immediately after marriage, regardless of whether they are physically and emotionally prepared [50–52]. It has also been argued that, in some cases, Jordanian women may use their ability to get pregnant soon after marriage as a way to raise their status and power among their in-laws. This provides insight on why FP methods are usually only considered after the first child has been born [53, 54].

Based on the WHOQOL-BREF scale, the overall mean (SD) scores of the four domains were found to be average, with the social relationships mean score being slightly higher than the other domains and the environmental domain being slightly lower.

Our results also revealed that women who used IUDs and women whose husbands used condoms as FP methods had better QoL in the four domains (physical health, psychological health, social relationships, and environment) than women who used OCs. In line with our findings, a recent prospective observational multicenter study conducted in Spain found that the Levonorgestrel intrauterine system (LNG-IUS) has a positive impact in terms of health related QoL. The results were obtained through conducting a SEC-QoL questionnaire at baseline (at the start of the LNG-IUS use) and 12 months afterwards. The study demonstrated improved scores in all domains of the SEC-QoL, including the social, menstrual symptoms, breast symptoms, psychological, and sexual domains. Beside its efficacy as a FP method, LNG-IUS provided women with additional non-contraceptive benefits (i.e. reduction in bleeding and menstrual pain) [21]. Similar results were reported by Singh from India [55].

Though IUDs may cause mild side effects which may interfere with a woman’s QoL, such as irregular bleeding, amenorrhea, and hormonal disturbances [56, 57], women who used IUDs and those whose husbands used condoms were nonetheless more satisfied than women who used OCs and sterilization. The use of female condoms is uncommon among Jordanian women, and 14.23% of the participating women in our study reported that their husbands used male condoms. Loss of sexual function and pleasure (among both males and females) is reported as being the most devastating side effect of using condoms [58, 59].

In consistence with our results, a recent randomized, double-blind, placebo-controlled trial which assessed the influence of a first-choice combined oral contraceptive on general well-being and depressive symptoms found that contraceptive pills are associated with reduced general well-being, self-control, and vitality among healthy women [19]. Also, in Japan, it has been reported that OCs can worsen QoL if used for contraceptive purpose only [60]. On the other hand, other studies have reported that OCs have no negative impact on QoL, especially among first time users [61]. Zhao et al. conducted a prospective cohort study on rural women who used OCs and IUDs in a Chinese province; the study found that oral pills and IUDs could significantly improve overall QoL [62]. The mechanism behind reduction in well-being by the use of OCs among healthy women is yet to be discovered. However, the decrease in free testosterone level induced by OCs use and the direct progestin-induced central nervous system effect may be the underlying causes behind reduced well-being [19].

Our results observed that women who had been using implant and injectable hormonal contraceptives have better QoL in terms of the physical health and social relationships domains. Di Carlo et al. studied the impact of contraceptive implants on QoL and sexual function among Italian women. Their results showed that general QoL and physical role status of the women improved significantly after use of the implant and that the implant had some positive effect on sexual function [63]. In a review of evidence on implant contraindications, satisfaction, and rate of continuation, Amico et al. found that whilst irregular bleeding was the most common reason for discontinuation of the implant, most of the participating women reported an overall reduction in bleeding. Additionally, complications related to its use, insertion, and removal were rarely reported [64]. Consistently, the Short Form-36 QoL questionnaire was employed to evaluate the HRQoL changes associated with the use of the depot medroxyprogesterone acetate (DMPA) injection. Along with its contraceptive efficacy, DMPA was linked with improvement in perceived physical health and with no adverse effects on mental health and sexual function [65].

To identify predictors of QoL for women using FP methods, multiple linear regression models were fitted to the scores of the four QoL domains. Common QoL predictors such as education level, family income, employment status and place of living were also identified as predictors of QoL for women using FP methods. Significant method specific predictors were also identified including birth spacing, experiencing side effects, and type of modern FP method. Regression results indicated that the more frequent side effects, the lower physical health. Women who used IUD as their FP method had higher probability of better physical QoL scores compared to women who used other methods. Those whose husbands used male condoms have shown higher probability of better QoL scores for the psychological, social relationships and environmental domains.

The multiple linear regression analysis confirmed that, in comparison with women who used oral pills, women who had chosen permanent sterilization as their contraceptive method had the lowest QoL scores in all of the four domains. This is not surprising given the adverse effects and negative consequences that tubal ligation can have on women’s overall wellbeing including psychological wellbeing and sense of productivity [66]. Similarly, Bahrami et al. adopted the (WHQOL-BREF) to compare the effects of different contraceptive methods on women’s QoL and found that women who had used sterilization or (DMPA) injectable contraceptive had the lowest level of QoL [67]. The logistic regression analysis extended our findings and showed that women who had undergone permanent sterilization were more likely to experience side effects and vaginal bleeding than women who used OCs. On the other hand, women who had been using other types of modern FP methods had less likelihood of experiencing side effects and bleeding. A study conducted on women who performed tubal ligation method found that one third of women perceived tubal ligation as a risky procedure that should not be performed, whereas the majority of women indicated that they will not advise women to undergo this procedure [68]. Another study revealed that depression significantly increased after tubal ligation due to several factors including changes in self-image, fear of negative perceptions of other people, and most importantly the husband’s negative attitude towards the procedure [69].

Because female sterilization or tubal ligation is a permanent contraceptive method which requires a one-time effort, some women still consider it to be the most convenient and effective FP method. In an Iranian study, many of the participating women stated that their main reason for choosing sterilization was to avoid the side effects of other contraceptive methods. However, the study results showed that the mean total scores of the SF-12 (Short Form Health Survey for evaluating QoL) and sexual function were significantly lower in the female sterilization group than in the non-sterilization group. 20% of the women in the sterilization group regretted their decision [26]. The main reason why these women had chosen permanent sterilization was that they felt fully satisfied with their family size [26]. However, the most widely known biological change after terminating fertility by tubal ligation is the post-sterilization syndrome, which comprises symptoms such as hormonal disturbance, menstrual irregularities, and vaginal bleeding [70–72]. This could explain why an increasing number of women are opting for reversible long-acting FP contraceptive methods rather than permanent tubal ligation [73].

Our multinomial logistic regression analysis showed that women who used IUDs and those who had undergone sterilization had a higher probability of experiencing vaginal bleeding than women who used OCs, yet women who used IUDs had the highest QoL (good/ very good). It has been argued that dissatisfaction due to bleeding profile is the most common reason for the discontinuation of a FP method [74]. Previous studies confirm that women who use IUDs report higher levels of satisfaction than users of other FP methods [75, 76]. Our analysis also demonstrated that women who used OCs and permanent sterilization were less satisfied with their health status than women who used other modern FP methods.

### Strengths and limitations

The current study has some limitations that need to be acknowledged. First, the sample collected through a cross-sectional household design from only two cities in Jordan; in which all eligible women were chosen based on a convenience frame. The convenience selectivity of the sample limits the generalizability of the findings as there is a possibility that women who agreed to participate in this study differed from those who refused to do so. For example, women who declined participation could have been unwilling to talk about their experiences with using modern FP methods or share their perceptions for several reasons that could directly impact QoL. Regardless, the authors tried to increase representativeness of the sample by stratifying each city into 5 sectors based on geographical location and density of population. Future research need to conduct a national survey using a randomization process to increase representativeness of the findings to a wider population.

Second, QoL was self-reported, and this might have been associated with false reporting. As with all self-reporting surveys, there is always a chance with inaccurate answers that over-estimate patients’ responses due to many reasons including courtesy bias [77], recall bias, and/or social desirability bias [77, 78]. However, the WHOQOL-BREF is a cross cultural, well validated, generic questionnaire that has been employed in various clinical conditions, including contraceptive use, in different countries worldwide [31, 35–37, 67]. Future studies need to use more objective measures to assess the impact of modern FP methods on women’s QoL, preferably using a longitudinal design to draw causal inferences. Additionally, Further research need to design and/or use more relevant questionnaires that truly reflect and measure the real perceptions of QoL among users of modern FP methods taking into considerations medical history and overall health status of women. Nonetheless, the authors tried to cover some of these aspects in this study by asking women specific questions related to FP methods and QoL such as birth spacing, the type of method used, duration of use, and previous information of the modern FP methods used, as well as previous knowledge of their side effects.

Finally, although we faced several difficulties in recruiting participants and in data collection, this study can be considered the first study that attempted to explore the association between modern FP methods and women’s QoL in Jordan.

### Implications

The current study revealed that the overall average scores of the four domains of QoL were relatively low among women who were using modern FP methods. This raises an important question about whether Jordanian women who are not using modern FP methods have the same low level of QoL. A national household survey in Jordan found that females reported poorer HRQoL in comparison with their counterpart males [79]. Thus, more attention to this segment of the population need to be provided to improve all aspects of QoL associated with reproductive health, not merely those related to FP issues. Similarly, more research need to be undertaken to compare QoL levels among Jordanian women who use either modern or traditional FP methods versus those who do not in order to establish a baseline for overall HRQoL among Jordanian women in general. Simple QoL questions could be used by health care professionals as a screening tool for overall health at every medical contact. Our findings also call for establishing programs to assess QoL among women in Jordan and other Arab countries focusing on the potential impact of modern FP use on overall QoL. These programs should be widely available for women who need it the most across the country.

However, women’s decisions about the best FP options are highly affected by the method’s efficacy, medical conditions, personal subjective experience with side effects or negative consequences, and the method’s impact on QoL [16, 80–82]. Jordanian women have some misconceptions in regards to the use and side effects of modern FP methods. Even worse, lack of trust between women and healthcare providers donates to the high percentages of discontinuation and/or reluctance to seek alternatives [53]. Hence, women who intend to use modern FP methods have the right to know the potential side effects and negative consequences of these methods, including emotional, psychological, physiological, and sexual consequences. Health care professionals, especially trained midwives, need to be willing to spend enough time with women, especially at maternal and child health medical centers, to increase their awareness about the realistic benefits and side effects of each of the modern FP methods in order to change some women’s misperceptions in this regard and ultimately increase their adoption of effective methods. This is of paramount importance in light of the findings of a recent national survey (2017/2018), which found that more than 60% of Jordanian women of childbearing age do not use any modern FP methods [29].

Further, the results of this study shed light on the importance of providing effective counselling and education on FP methods to women of reproductive age in Jordan, and underlines a need to provide couples with premarital counselling on FP and potential risks associated with early pregnancy, particularly in rural areas with high fertility rates. Importantly, clinicians need to use a patient-centred approach when providing counselling about all FP methods and support women’s reproductive autonomy [64, 83]. This can be achieved by providing and securing various alternatives for women in regards to modern FP methods especially those that are perceived as well tolerated, highly effective in controlling blood loss, such as IUD and definitely a better alternative to hysterectomy for women with heavy menstrual bleeding [55].

The current study also found that women who used IUDs and women whose husbands used condoms were nonetheless more satisfied and had better QoL than women who used OCs and sterilization. Studies show that women who encounter complications while using a certain contraception method tend to have a lower QoL. Similarly, women who experienced side effects reported lower QoL scores than those who did not [84]. In the case of condom use, it is less likely that women experience side effects thus they tend to be more satisfied. Indicating a need for designing and implementing specific interventions, as a part of counseling practice, that teach women ways to cope with side effects and/or complications with various modern FP methods and evaluating the impact of these methods on both QoL and sexual life [84].

The relatively higher QoL scores observed among women whose husbands used condoms raising the issue of male participation in the use of contraception. A study conducted on male Muslims in a Ghanaian municipality concluded that condom is the most preferred and used modern contraceptive, suggesting that male Muslims need to be motivated to discuss FP with friends and wives, as well as programs should be developed to focus on involving men in the decision of FP with women [85]. This is very important given the fact that sociocultural factors could play a vital role in male involvement in FP. Therefore, social support, adequate information, and shared responsibility can all motivate men to have a more vital role in FP and use of certain contraceptive methods. Additionally, increasing literacy of reproductive health among men to improve their attitude and change their perceptions to better participate in reproductive health services is needed [86, 87]. Male participation in FP, in turn, enhances the status of women. A qualitative study on Iranian Muslim women revealed that participants felt more empowered when shared FP decisions were made with their male spouses based on agreement and support [88]. Consequently, more attention is needed to underpin strategies to encourage gender equity, shared decision making, shared responsibility and actual participation of men, empowering women, and to optimize worth of male participation [89].

## Conclusion

Overall, participating women reported relatively poor quality of life particularly those using oral contraceptive bills in comparison to those who use IUDs and male condoms. When comparing QoL across different methods of modern contraceptives, we found that women who used oral contraceptives and those who had undergone permanent tubal sterilization were likely to have a lower level of QoL than users of IUDs and implant and injectable hormones and women whose husbands used condoms. Also, women who used oral contraceptives and women who had undergone tubal sterilization were less likely to be satisfied and had the highest probability of frequent bleeding. Being able to decide freely whether and when to have children is regarded as a fundamental reproductive health right and is strongly linked to women’s health and quality of life.

## Data Availability

The datasets generated and analysed during the current study are included in this manuscript article (Tables 1, 2, 3, 4 and 5).

## Abbreviations

DMPA: Depot medroxyprogesterone acetate
FP: Family planning
FR: Fertility rate
HRQoL: Health-related quality of life
IER: Information, Evidence, and Research
IUDs: Intra Uterine Devices
JD: Jordanian Dinar
KAUH: King Abdullah University Hospital
LNG-IUS: Levonorgestrel intrauterine system
OCPs: Oral contraceptive pills
OCs: Oral contraceptives
PGWBI: Psychological General Well-Being Index
QoL: Quality of life
SD: Standard deviation
SEC-QOL: Spanish Society of Contraception Quality-Of-Life
SF-12: Short Form Health Survey for evaluating QoL
TFR: Total Fertility Rate
WHO: World Health Organization
WHOQOL-BREF: World Health Organization Quality of Life-BREF
